# Exploring the Genetic Diversity of *Candida albicans* in Animal Hosts: A One Health Perspective

**DOI:** 10.1111/zph.70083

**Published:** 2026-08-10

**Authors:** Lucilla Lo Re, Silvia Crotti, Letterio Giuffrè, Andrea Bonomo, Deborah Cruciani, Alice Ranucci, Ida Pernice, Carla Lo Passo, Giuseppe Criseo, Orazio Romeo

**Affiliations:** ^1^ Dipartimento di Scienze Chimiche, Biologiche, Farmaceutiche ed Ambientali Università di Messina Messina Italy; ^2^ Istituto Zooprofilattico Sperimentale dell'Umbria e delle Marche “Togo Rosati” (IZSUM) Perugia Italy; ^3^ Dipartimento di Matematica Università di Pavia Pavia Italy

**Keywords:** animal host, *Candida albicans*, genetic diversity, genotyping, *hwp1*‐gene, MLST

## Abstract

**Introduction:**

*Candida albicans* is an important priority fungal pathogen that commonly colonizes humans and a wide range of animal hosts, although its population structure in wildlife remains poorly explored.

**Methods:**

In this study, 41 *C. albicans* isolates obtained from Italian wild animals (European hedgehogs, wolves, foxes, badgers, roe deer and porcupine) were identified by *hwp1*‐specific PCR and subsequently genetically characterized using ABC typing, mating‐type locus analysis and multilocus sequence typing (MLST).

**Results:**

Several isolates were heterozygous at the *hwp1* locus, and one rare 850 bp homozygous variant was detected in animals for the first time. ABC typing assigned 27 isolates to genotype B, 10 to genotype A and 4 to genotype C. All isolates were heterozygous (a/α) at the mating‐type locus. MLST analysis identified 116 alleles, including 20 novel alleles, and 36 diploid sequence types, of which 28 were newly described. Twenty‐two isolates clustered within 11 clonal complexes shared with human isolates, whereas 19 were singletons. Animal isolates were distributed across nine established MLST clades, with no clear association with host species. However, nearest‐neighbour analysis revealed partial but significant genetic segregation between human and animal isolates, suggesting a degree of host‐associated population structuring.

**Conclusions:**

Overall, these findings indicate that animal‐associated *C. albicans* populations represent a substantial and largely unexplored reservoir of genetic diversity and contribute to a better understanding of the population structure and ecology of this opportunistic pathogen within a One Health framework.

## Impacts

- This study describes an extensive genetic diversity in wildlife‐associated *Candida albicans* isolates.
- Several MLST genotypes were shared between human and animal *C. albicans* populations.
- This study provides evidence for interspecies transmission of *C. albicans* among humans and animals.

## 1 Introduction

In recent decades, the burden of fungal diseases has increased dramatically, emerging as a major global public health concern. The global prevalence of invasive fungal infections is now estimated at approximately 6.5 million cases annually, resulting in more than 2.5 million deaths worldwide (Denning 2024). Notably, invasive infections caused by *Candida* species affect approximately 1.5 million individuals each year, with nearly one million associated deaths, underscoring the high mortality and substantial clinical impact of these infections (Denning 2024).

Beyond their well‐recognized impact on humans, fungal infections also represent a critical and often underappreciated threat to animal health. The prominent role of certain fungal pathogens, such as *Candida albicans* , in infections affecting both humans and animals highlights the interconnected nature of fungal pathogenesis across species and emphasizes the importance of addressing diseases caused by this pathogen within the One Health framework that intrinsically links human, animal and environmental health (Woods et al. 2023). Today, zoonotic infections have emerged as an escalating global health concern, given that nearly 75% of emerging infectious diseases originate from animals and involve a wide range of etiological agents, including viruses, bacteria, parasites and fungi (Adebiyi and Oluwayelu 2018). According to the World Health Organization (WHO), zoonoses are diseases naturally transmitted from vertebrate animals to humans, and more than 70% of emerging zoonotic diseases are associated with wildlife, which constitutes a significant reservoir of clinically relevant fungal pathogens (Carpouron et al. 2022). *C. albicans* , for example, has been recovered from a wide range of animal species, including mammals such as dogs, cats, horses, cattle, dromedaries and wild boar (Willems et al. 2017; Dalvand et al. 2018; Osman et al. 2019; Fayez et al. 2022; Fayed et al. 2025; Rhimi et al. 2025), birds, including wild and domestic species such as geese, hawks, ducks, ostriches, laying hens and other poultry (Liu et al. 2018; Domán et al. 2021, 2023; Rhimi et al. 2025) and reptiles, including exotic species and lizards (Wrobel et al. 2008; Rhimi et al. 2025). This species remains the leading cause of invasive candidiasis in humans (Pfaller et al. 2019) and has recently been designated as a critical‐priority fungal pathogen by the WHO (Casalini et al. 2024). Genetically, *C. albicans* strains of animal origin show considerable variability, with some genotypes also found in humans, suggesting potential zoonotic transmission (Domán et al. 2024; Osman et al. 2019; Rhimi et al. 2025). However, despite the increasing number of animal isolates reported, genetic data on wildlife‐derived *C. albicans* remain scarce.

According to the most recent data from the international *C. albicans* MLST database (https://pubmlst.org/organisms/candida‐albicans; last accessed 20 December 2025), only 239 of the 5841 (~4.1%) genotyped strains are of animal origin, and only 17 originate from the Italian fauna. This limited representation underscores the need to further investigate the intra‐species and inter‐host genetic variability of *C. albicans* (Dalmieda and Xu 2026), particularly from a One‐Health perspective.

In this study, we genetically characterized *C. albicans* isolates from wild animals in Italy aiming to investigate their genetic diversity and population structure in a veterinary context. Using multilocus sequence typing (MLST) and other molecular approaches, we assessed intra‐species variation and compared genotypes of animal‐derived isolates with those of human origin currently available in the MLST database, providing new insights into the genetic diversity, population structure and host‐associated distribution of this important One Health fungal pathogen.

## 2 Materials and Methods

### 2.1 Yeast Isolates, Phenotypic and Molecular Identification

A total of 41 *C. albicans* isolates were examined in this study (Table S1). The fungal isolates were collected through oral swabs (*n* = 28), rectal swabs (*n* = 9), oropharyngeal swabs (*n* = 3) and spines (*n* = 1) from different wild animal species like European hedgehog (*n* = 19), wolf (*n* = 8), fox (*n* = 7), badgers (*n* = 5), roe deer (*n* = 1) and porcupine (*n* = 1) (Table S1). Samples were collected within the framework of a passive wildlife surveillance program conducted by the Istituto Zooprofilattico Sperimentale dell'Umbria e delle Marche ‘Togo Rosati’ (IZSUM, Italy), involving deceased wild animals submitted for post‐mortem examination. Biological samples were collected during routine necropsies performed for diagnostic investigations. All *C. albicans* isolates were regarded as colonizing yeasts rather than the etiological agents of disease, since no clinical or pathological evidence of candidiasis was observed in the examined animals.

For primary fungal isolation, swabs were streaked onto Sabouraud dextrose agar (SDA) and incubated aerobically at 37°C for 48–72 h. Yeast colonies were then subcultured onto CHROMagar *Candida* (CHROMagar, France) for direct detection and presumptive identification of *C. albicans* , based on the characteristic green coloration of its colonies.

Final identification was performed using a species‐specific PCR‐based method targeting the hyphal wall protein 1 (*hwp1*) gene, which allows discrimination of *C. albicans* from its phylogenetically closest relatives, *C. dubliniensis* and *C. africana* (Romeo and Criseo 2008).

Total genomic DNA was extracted using the high‐speed glass bead‐beating method combined with the conventional phenol‐chloroform‐isoamyl alcohol extraction and ethanol precipitation (Müller et al. 1998). A schematic overview of the molecular workflow adopted in this study is shown in Figure 1.

**FIGURE 1:**
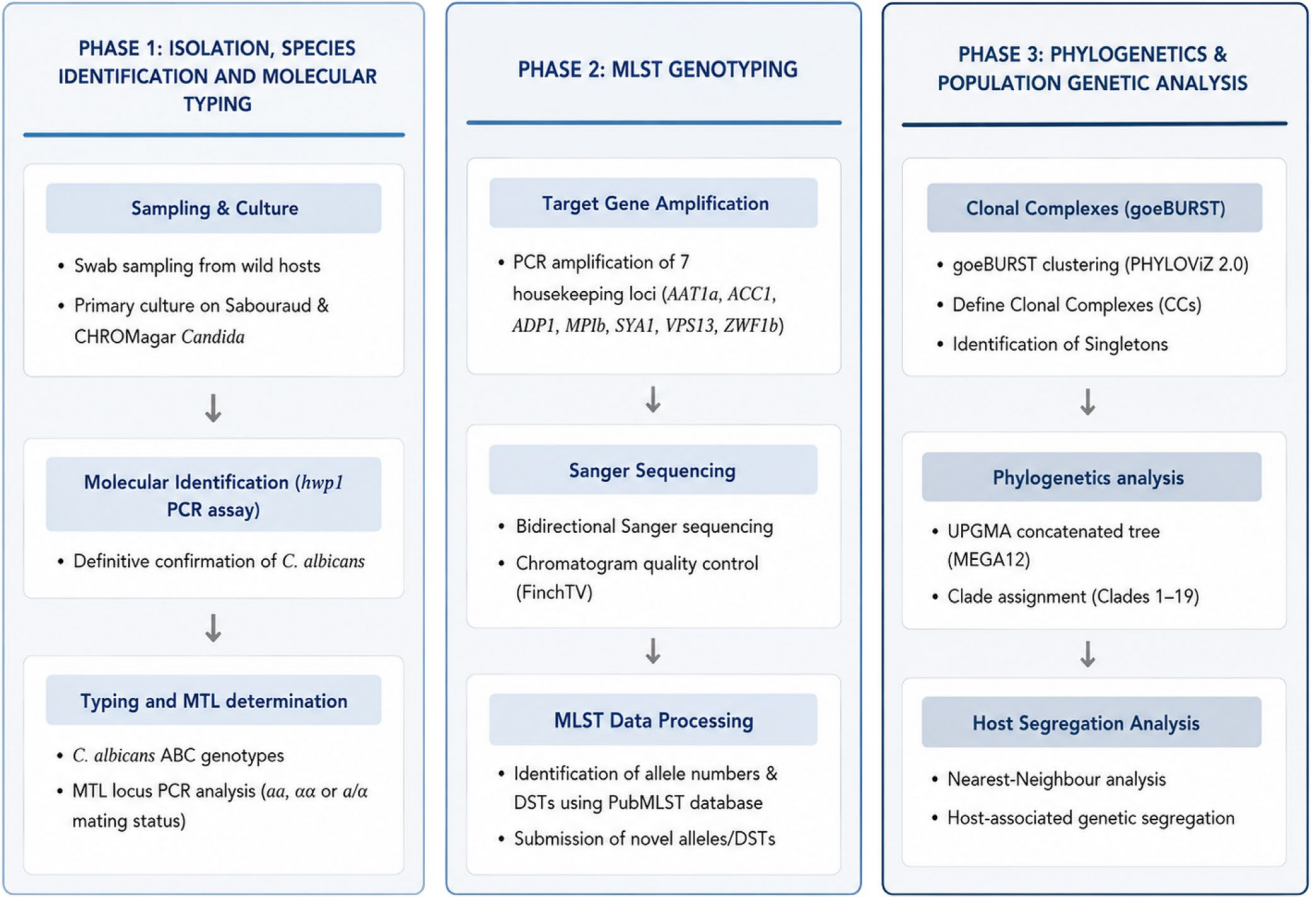
Schematic workflow illustrating the integrated molecular and population genetic approaches used in this study.

### 2.2 ABC Genotyping and Mating‐Type Locus Characterization

Genetic variability among *C. albicans* isolates recovered in this study was first assessed using the PCR‐based ABC typing method which classifies isolates in five different genotypes based on the number and size of the amplicons produced: genotype A (~450 bp), genotype B (~840 bp), genotype C (~450 bp and ~840 bp), genotype D (~1080 bp; now recognized as *C. dubliniensis*) (McCullough et al. 1999) and genotype E (~962 bp) (Tamura et al. 2001).

The distribution of mating types among animal *C. albicans* isolates was assessed by PCR amplification of the mating type‐like loci *MTLα1*, *MTLa1* and *MTLa2*, according to Hull and Johnson (1999).

### 2.3 Genotyping of *C. albicans* by Multilocus Sequence Typing (MLST) Analysis

Genotyping of animal *C. albicans* isolates was carried out using the officially adopted MLST scheme, targeting seven housekeeping genes: *AAT1a*, *ACC1*, *ADP1*, *MPIb*, *SYA1*, *VPS13* and *ZWF1b* (Bougnoux et al. 2003). For each isolate, internal fragments of the seven MLST loci were individually amplified using DreamTaq Green PCR Master Mix (Thermo Fisher Scientific, Milan, Italy), 50 ng of genomic DNA and 0.2 μM of each locus‐specific MLST primer, following previously described protocols (Bougnoux et al. 2002; Tavanti et al. 2003). PCRs were performed in a MyCycler T100 thermocycler (Bio‐Rad, Milan, Italy) with an initial denaturation at 95°C for 3 min, 35 cycles of 95°C for 30 s, 55°C for 1 min and 72°C for 45 s, followed by a final extension at 72°C for 10 min. PCR products were analysed by 1.8% (w/v) agarose gel electrophoresis to verify the presence and size of the expected amplicons, and subsequently purified using the QIAquick PCR Purification Kit (Qiagen, Italy) before bidirectional sequencing at Eurofins Genomics, Ebersberg, Germany (www.eurofinsgenomics.eu) via standard Sanger sequencing with the same MLST locus‐specific primers used for PCR.

Each forward and reverse sequencing trace was visually inspected using FinchTV v1.4 (GeoSpiza Inc., Seattle, WA) to identify base‐calling errors and potential heterozygous polymorphisms. Nucleotide sequences, including single‐nucleotide polymorphisms (SNPs), were annotated in text‐based FASTA format using the International Union of Pure and Applied Chemistry (IUPAC) single‐letter nucleotide code. DNA sequences from each MLST locus were then compared with the corresponding reference sequences in the central *C. albicans* PubMLST database (https://pubmlst.org/organisms/candida‐albicans) to assign allele numbers and define the diploid sequence type (DST) for each isolate.

Unassigned allele sequences were submitted to the official PubMLST database, where new allele numbers, including novel DSTs, were assigned by the database curator, Prof. Marie‐Elisabeth Bougnoux.

### 2.4 MLST‐Based Phylogeny and Population Structure of *C. albicans*

Phylogenetic relationships were inferred among the 41 animal isolates analysed in this study, along with an additional 10–15 DSTs representative of each of the 19 *C. albicans* clades described by Gong et al. (2018). For each *C. albicans* isolate, nucleotide sequences were concatenated in the order of loci specified by the MLST scheme (*AAT1a*, *ADP1*, *ACC1*, *MPIb*, *SYA1*, *VPS13*, *ZWF1b*), generating a contiguous sequence of 2883 bp, and edited as described by Odds et al. (2007). A phylogenetic tree was then constructed using the unweighted pair‐group method with arithmetic mean (UPGMA) in MEGA version 12 (Kumar et al. 2024), employing the p‐distance model and 1000 bootstrap replicates.

Finally, genotype clustering analysis was performed to predict the clonal complexes (CCs) of the animal isolates recovered in this study, together with an additional 5841 *C. albicans* isolates retrieved from the international PubMLST database (last accessed 20 December 2025), representing 3678 unique DSTs. The analysis was conducted using the global optimal eBURST (goeBURST) algorithm implemented in PHYLOViZ 2.0 software (Nascimento et al. 2017). CCs were assigned based on single‐locus variant (SLV) analysis, grouping isolates that differed at only one of the seven MLST loci into the same complex.

### 2.5 Assessing Genetic Separation Between Human and Animal Isolates Using Nearest‐Neighbour Analysis

To test whether *C. albicans* isolates of human origin mix randomly with animal‐derived isolates (i.e., no genetic separation) or whether there is evidence of host‐driven genetic segregation, a pairwise genetic distance and SNP‐based matrix were generated using MEGA12. A nearest‐neighbour (NN) analysis was then applied to test the hypothesis of genetic segregation between host‐associated populations, following the statistical framework described by Jacobsen et al. (2008).

To minimize the potential confounding effect of geographic variation, the analysis was restricted to isolates from Italy. The final MLST dataset comprised 78 *C. albicans* isolates from human and animal origin, representing all eligible Italian isolates available at the time of analysis. These included 36 animal‐derived isolates characterized in the present study, 27 previously published Italian animal isolates (Rhimi et al. 2025) and 15 Italian human clinical isolates retrieved from the PubMLST database. To avoid overrepresentation of identical genotypes, only unique DSTs were retained. A Neighbour‐Joining tree was then constructed using the Kimura two‐parameter model with 1000 bootstrap replicates.

The statistical significance of the segregation was evaluated using a binomial probability test. Under the null hypothesis of random mixing (panmixia), the probability (*p*) that the nearest neighbour of any given isolate would be an animal isolate was calculated based on the frequency of animal isolates in the remaining dataset, according to the formula: $p=N_{\text{animal}}/\left(N_{\text{total}}-1\right).$ The observed number of human isolates with an animal nearest neighbour was then compared to the expected number under the null hypothesis, which assumes no genetic separation between human and animal isolates. A *p*‐value < 0.05 was considered statistically significant, indicating that human isolates are genetically closer to other human isolates than expected by chance, consistent with host‐specific adaptation or segregation.

## 3 Results

### 3.1 Fungal Isolates, Phenotypic and Molecular Identification

All fungal isolates included in this study produced green colonies on CHROMagar *Candida*, characteristic of *C. albicans* . Partial amplification of the *hwp1* gene generated the expected 941 bp amplicon in 40 isolates, of which 12 (29.3%) also yielded an additional fragment of approximately 850 bp, indicating heterozygosity at this locus (Table S1). Isolate IZSUM‐7 produced a single amplicon of ~850 bp, which corresponds to a rare homozygous *hwp1* pattern recently identified in some Colombian vulvovaginal *C. albicans* isolates (Morales‐López et al. 2025).

### 3.2 Genotyping and Genetic Diversity of *C. albicans* Isolates

ABC typing showed that most *C. albicans* isolates analysed in this study belonged to genotype B (27/41; 65.8%) (Table S1). These isolates were detected in all sampled animal species, with the European hedgehog showing the highest number of B isolates (13/27; 48.1%), followed by the wolf (6/27; 22.2%), the badger and fox (3/27; 11.1% each) and the porcupine and roe deer (1/27; 3.7% each) (Table S1). Genotype A was identified in 10 isolates (10/41; 24.4%), recovered from hedgehogs (3/10; 30%), foxes (3/10; 30%), and wolves and badgers (2/10; 20% each) (Table S1). Only four isolates (4/41; 9.8%), three from hedgehogs (3/4; 75%) and one from fox (1/4; 25%), were classified as genotype C. In addition, all animal isolates included in this study were heterozygous (a/α) at the mating‐type‐like (*MTL*) locus (Table S1).

Sequencing of internal fragments (373–491 bp) from all seven loci included in the MLST scheme yielded 2883 aligned nucleotides per fungal isolate, allowing the identification of 116 distinct alleles. Of these, 20 (17.2%) were novel and were submitted to the *C. albicans* PubMLST database.

The *SYA1* locus exhibited the highest allelic diversity, with 22 alleles identified, whereas *AAT1a* was the least diverse locus, with 13 alleles (Table 1). Overall, 92 polymorphic sites were detected across all MLST loci, most of which were located in *SYA1* (20 sites; 21.7%), followed by *VPS13* (18; 19.6%), *MPIb* (15; 16.3%), *ADP1* (13; 14.1%), *ZWF1b* (10; 10.9%), *ACC1* (9; 9.8%) and *AAT1a* (7; 7.6%) (Table 1).

**TABLE 1:** Allelic diversity and polymorphisms of the seven MLST loci analysed in this study.

MLST locus	Sequenced fragment size (bp)	N° of alleles	N° of variable sites	Nucleotide position
*AAT1a*	373	13	7	7, 28, 40, 70, 89, 124, 325
*ACC1*	407	14	9	8, 82, 119, 211, 281, 282, 317, 392, 393
*ADP1*	443	15	13	17, 35, 40, 46, 109, 125, 166, 205, 225, 232, 352, 361, 391
*MPIb*	375	16	15	21, 27, 34, 36, 66, 72, 88, 107, 193, 234, 237, 276, 289, 307, 318
*SYA1*	391	22	20	1, 25, 48, 50, 61, 89, 100, 125, 142, 149, 160, 185, 215, 248, 296, 304, 307, 313, 322, 351
*VPS13*	403	19	18	33, 49, 116, 134, 194, 212, 217, 241, 266, 281, 282, 320, 322, 326, 328, 338, 370, 375
*ZWF1b*	491	17	10	23, 31, 43, 49, 55, 70, 274, 337, 379, 482
Total	2.883	116	92	

When combined, the MLST alleles yielded a total of 36 unique DSTs, of which 28 (28/36; 77.8%) were novel and were submitted to the *C. albicans* MLST database (Table 2). The most frequently identified genotype was DST 1109 (5/41 isolates; 12.2%), followed by DST 3671 (2/41; 4.9%), while each of the remaining 34 DSTs was detected only once (1/41; 2.4% each) (Table 2). Most isolates with DST 1109 were obtained from oral swabs of European hedgehogs (3/5; 60%), with the remaining isolate originating from a wolf and porcupine respectively (1/5; 20% each).

**TABLE 2:** *Candida albicans* allelic profiles, DSTs and CCs identified by MLST analysis.

Isolate code	MLST loci							DST	CC	CLADE	ABC‐typing
	*AAT1a*	*ACC1*	*ADP1*	*MPIb*	*SYA1*	*VPS13*	*ZWF1b*				
IZSUM‐1	21	17	21	19	53	83	22	1646	7	12	B
IZSUM‐2	13	10	15	6	7	37	137	4235^a^	4	3	B
IZSUM‐3	3	5	5	2	275^a^	25	5	4240^a^	S	1	A
IZSUM‐4	13	3	4	6	276^a^	20	18	4241^a^	39	15	C
IZSUM‐5	31	2	5	2	277^a^	20	5	4242^a^	S	1	A
IZSUM‐6	5	2	5	2	2	20	5	4236^a^	1	1	A
IZSUM‐7	59	34	15	1	60	20	70	1109	78	11	B
IZSUM‐8	59	34	15	1	60	20	70	1109	78	11	B
IZSUM‐9	74	34	15	1	278^a^	20	70	4244^a^	S	11	B
IZSUM‐10	4	7	16	110	13	19	14	1737	16	10	B
IZSUM‐11	74	7	16	110	13	358^a^	14	4246^a^	16	10	B
IZSUM‐12	59	34	15	1	60	20	70	1109	78	11	B
IZSUM‐13	80	3	6	4	279^a^	45	13	4249^a^	23	14	B
IZSUM‐14	59	5	21	2	80	362^a^	15	4250^a^	13	17	B
IZSUM‐15	28	5	21	2	80	108	348^a^	4252^a^	S	17	B
IZSUM‐16	59	34	15	1	60	20	70	1109	78	11	B
IZSUM‐17	152	138^a^	15	1	60	20	349^a^	4254^a^	S	11	B
IZSUM‐18	13	3	4	6	280^a^	20	18	4255^a^	39	15	C
IZSUM‐19	3	3	259^a^	4	27	47	13	4256^a^	14	14	B
IZSUM‐20	6	3	21	4	27	47	13	3671	14	14	B
IZSUM‐21	13	3	4	6	34	20	18	172	39	15	C
IZSUM‐22	59	34	15	1	60	20	70	1109	78	11	B
IZSUM‐23	13	3	260^a^	227^a^	281^a^	20	18	4257^a^	S	15	C
IZSUM‐24	3	3	37	2	38	46	12	811	18	7	A
IZSUM‐25	2	3	2	2	2	6	5	73	1	1	A
IZSUM‐26	74	34	15	1	60	363^a^	70	4261^a^	S	11	A
IZSUM‐27	3	2	5	149	282^a^	20	5	4262^a^	S	1	B
IZSUM‐28	21	26	25	9	38	65	12	3126	62	7	A
IZSUM‐29	6	3	21	4	27	47	13	3671	14	14	B
IZSUM‐30	3	3	21	4	27	47	13	4237^a^	14	14	B
IZSUM‐31	6	5	21	2	335^a^	108	271	4320^a^	S	17	B
IZSUM‐32	3	139^a^	21	50	53	47	164	4323^a^	S	14	B
IZSUM‐33	3	8	25	9	76	47	12	4287^a^	S	7	B
IZSUM‐34	6	98	21	8	116	108	68	4288^a^	S	S	B
IZSUM‐35	3	98	6	14	53	112	12	4289^a^	S	1	A
IZSUM‐36	3	26	23	38	53	53	12	4290^a^	S	1	A
IZSUM‐37	3	11	137	95	76	32	189	4291^a^	S	3	B
IZSUM‐38	3	7	16	7	147	165	14	4292^a^	S	10	B
IZSUM‐39	3	34	13	1	336^a^	20	101	4324^a^	S	11	B
IZSUM‐40	3	47	96	1	336^a^	20	35	4325^a^	S	11	B
IZSUM‐41	3	26	5	17	335^a^	58	68	4326^a^	S	1	A

Abbreviations: CC, clonal complex; DST, diploid sequence type; S, singleton.

^a^ New alleles/DSTs detected in this study.

To explore the evolutionary relationships among *C. albicans* genotypes, MLST data from the present study were analysed together with 3678 distinct DSTs deposited in the PubMLST database using the goeBURST algorithm, which clusters closely related DSTs into clonal complexes based on single‐locus variants. This analysis grouped the complete dataset into 184 CCs and 1229 singletons (S). Among the 41 animal isolates of this study, 22 (22/41; ~54%) clustered within 11 clonal complexes (CCs 1, 4, 7, 13, 14, 16, 18, 23, 39, 62 and 78) (Figure 2), whereas the remaining 19 isolates (19/41; 46%) were classified as singletons (Table 2).

**FIGURE 2:**
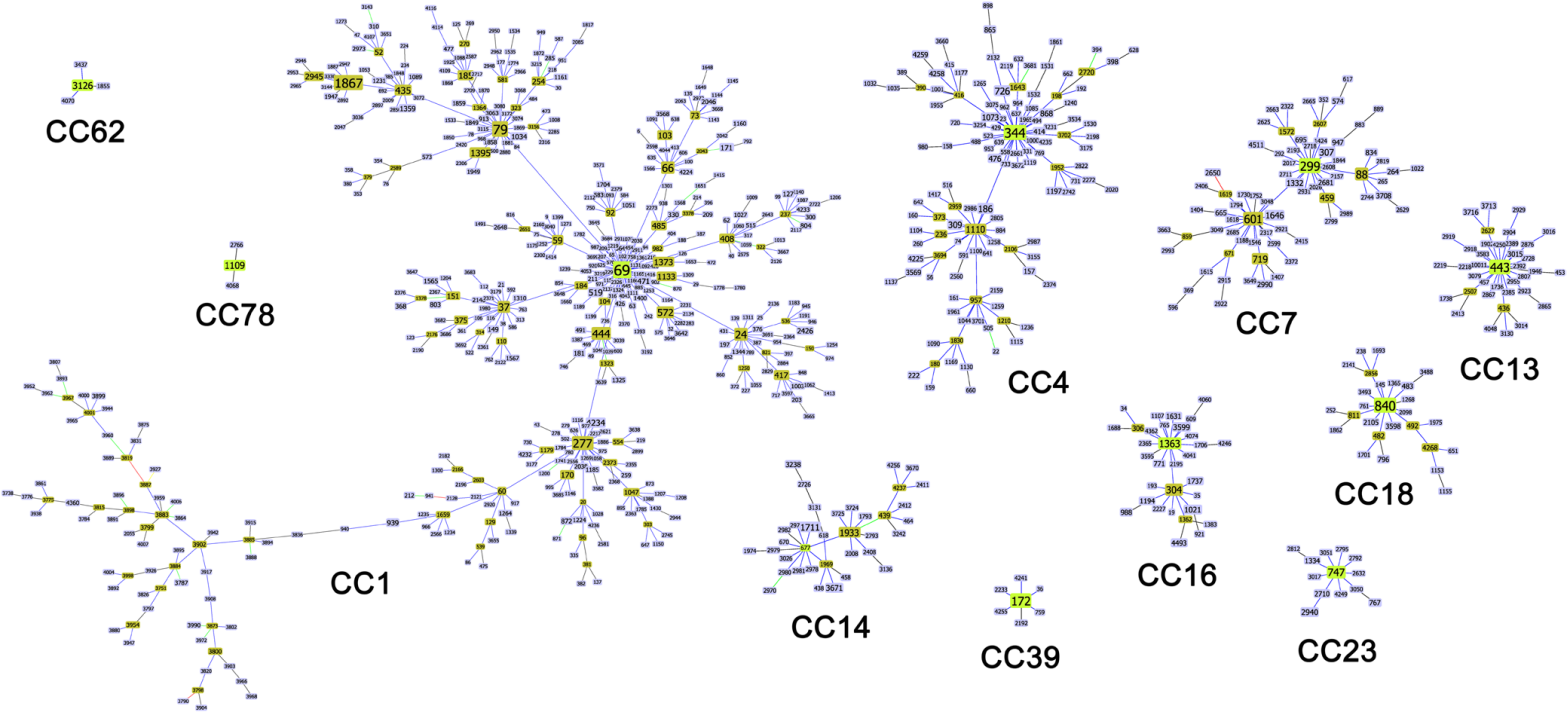
Details of the 11 goeBURST clonal complexes detected in this study. For each cluster, the total number of DSTs is shown. The presumed founder genotypes (in green) are positioned at the center of each CC, while the founders of the subgroups are shown in olive green. The size of each rectangle containing the DST code reflects the number of isolates with that MLST genotype in the entire dataset used for the analysis. The lengths of the lines are not significant.

The CC‐78 contained the largest number of isolates from this study (5/41), all sharing the founder genotype DST 1109 (Table 2), which is genetically related to two distinct DSTs originating from an oropharyngeal sample of a Chinese patient with rheumatoid arthritis (DST 2766) and from the faeces of an Italian wild boar (DST 4068) (Figure 2) (Rhimi et al. 2025).

An interesting clonal group is CC‐39, which contains a relatively higher proportion of animal‐derived isolates (15 animals and 9 human). This cluster also includes three of the four isolates classified as genotype C according to the ABC typing method used in this study: IZSUM‐4 (DST 4241), IZSUM‐18 (DST 4255) and IZSUM‐21 (DST 172) (Table 2). DSTs 4241 and 4255 are two newly identified animal‐associated genotypes, whereas several DST 172 isolates of the CC‐39 have previously been reported from avian and human samples in the PubMLST database.

Animal‐derived *C. albicans* isolates also predominate in CC‐62, which comprises four distinct MLST genotypes (DSTs 1855, 3126, 3437 and 4070) recovered from French birds, Italian wild boar and human samples. Within this cluster, the DST 3126 genotype was isolated from both animal (birds) and human (vaginal swab) sources. Additional *C. albicans* genotypes shared between human and animal samples were detected in CC‐1 (DST 73), CC‐7 (DST 1646) and CC‐18 (DST 811); however, these clonal complexes were predominantly composed of human isolates from different body sites.

Phylogenetic analysis of our fungal isolates, together with several reference DSTs representative of the 19 MLST clades of *C. albicans* described by Gong et al. (2018), showed that the 41 isolates analysed were distributed across nine distinct clades (1, 3, 7, 10, 11, 12, 14, 15 and 17) and one singleton (isolate IZSUM‐34) (Table 2; Figure 3). Most isolates clustered in clade 11 (10/41; ~24.4%), followed by clade 1 (8/41; 19.5%) and clade 14 (6/41; 14.6%). Smaller proportions were assigned to clade 15 (4/41; 9.75%), clades 7, 10 and 17 (3/41 each; 7.3%), clade 3 (2/41; ~4.9%) and clade 12 (1/41; 2.4%) (Table 2; Figure 3). European hedgehog isolates, the most frequent in this study (19/41; 46.3%), were present in all clades except 7 and 17, which contained only isolates from badgers, foxes and wolf. Notably, all four *C. albicans* genotype C isolates were exclusively clustered in clade 15 (Table 2; Figure 3), whereas 9 of 12 *hwp1*‐heterozygous *C. albicans* isolates grouped in clade 11.

**FIGURE 3:**
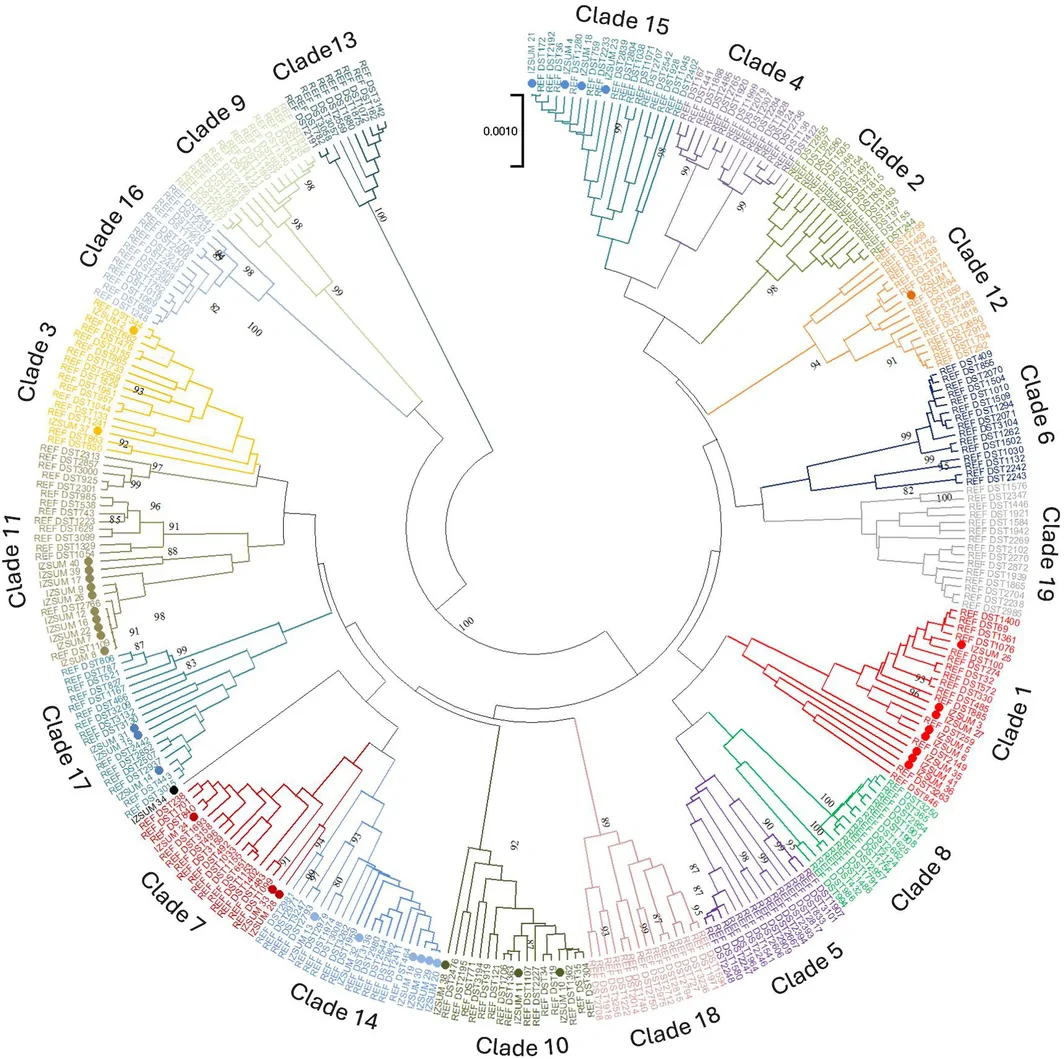
UPGMA phylogenetic tree showing the relationships among the 41 *C. albicans* isolates analysed in this study and several animal and human reference isolates representing the 19 MLST clades of *C. albicans* described by Gong et al. (2018). Numbers at the cluster nodes indicate bootstrap support values. Dots denote the *C. albicans* isolates genotyped in this study.

No specific correlation was observed between phylogenetic clades and host species (Figure 3).

However, nearest‐neighbour analysis of a subset of MLST data restricted to italian *C. albicans* isolates revealed a certain degree of genetic separation between human and animal isolates. Using this dataset, which comprises a total of 78 Italian *C. albicans* isolates (63 from animals and 15 from humans), under the null hypothesis of no genetic separation, the probability that a human isolate's closest relative is an animal isolate would be determined solely by the ratio of animal isolates to the total number of remaining isolates (63/77). By multiplying this probability (0.81) by the number of Italian human isolates in the dataset (*n* = 15), we calculated that in 12 cases a human isolate would be expected to have an animal isolate as its closest neighbour. However, based on Neighbour‐Joining phylogenetic analysis (Figure 4), we observed that only 8 of 15 human isolates had an animal isolate as closest neighbour, indicating partial but not complete host‐associated genetic structuring (binomial test, *p*‐value = 0.01). This genetic segregation can also be visually observed in the N‐J tree, which shows distinct clustering of human and animal isolates (Figure 4). Comparable results were observed when NN analysis was applied to the SNP‐based matrix, with 7 human isolates having an animal isolate as their nearest neighbour (binomial test, *p* = 0.002).

**FIGURE 4:**
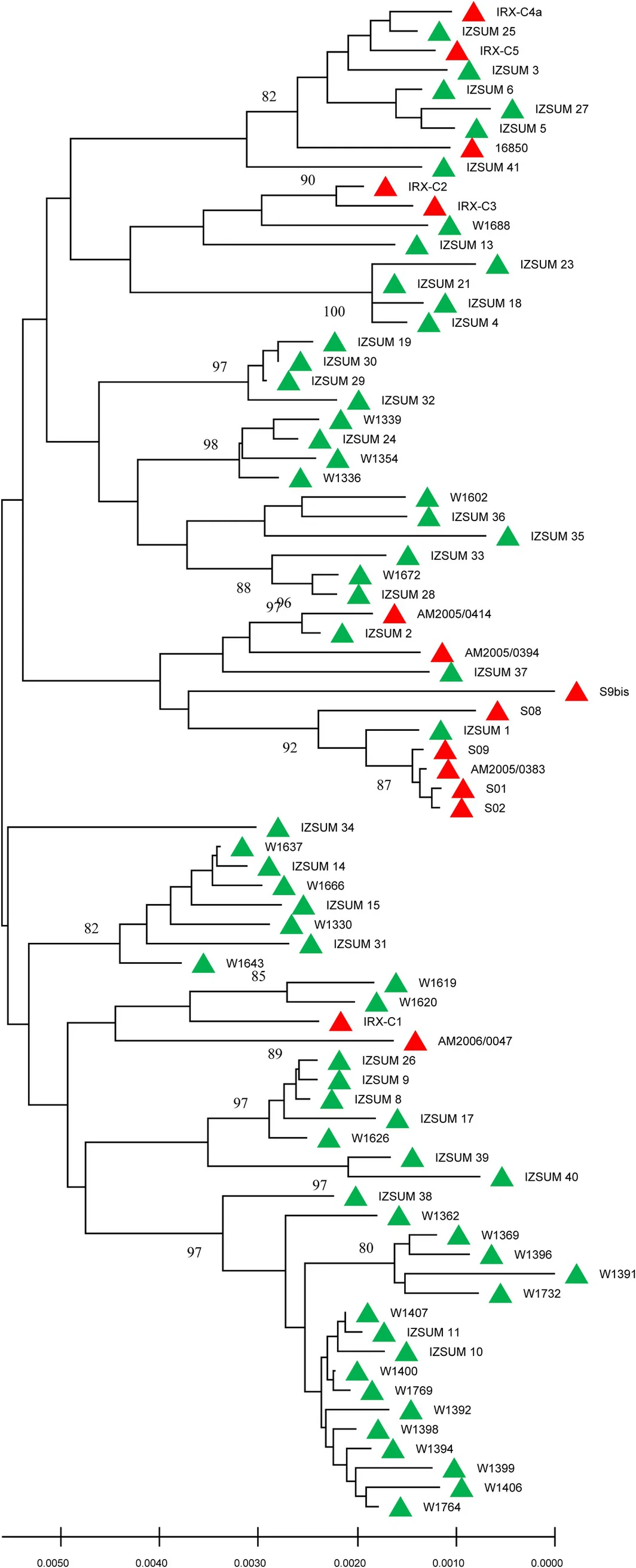
Neighbour‐Joining tree of 78 Italian *C. albicans* isolates of human and animal origin. The tree was constructed using the Kimura two‐parameter model with 1000 bootstrap replicates. The dataset includes 15 human‐derived isolates retrieved from PubMLST (red triangles) and 63 animal‐derived isolates (36 identified in this study and 27 reported by Rhimi et al. 2025; green triangles).

## 4 Discussion

*C. albicans* is a ubiquitous opportunistic WHO‐priority pathogen that normally colonizes the microbiomes of humans and a wide range of animal hosts. However, its ecology and population structure in wild animals and/or in veterinary contexts remain poorly explored, as evidenced by the strong bias of the international PubMLST database (pubmlst.org/organisms/candida‐albicans; last accessed 10 February 2026) toward human‐derived isolates (3961 human vs. 267 animal isolates).

Investigating the genetic diversity of *C. albicans* in animals is therefore essential to elucidate its evolutionary dynamics and the interplay among human, animal and environmental reservoirs, in line with a One Health perspective.

In this study, we genetically characterized 41 *C. albicans* isolates obtained from Italian wild animals and revealed a highly heterogeneous population structure, with a high proportion of novel MLST genotypes (~77.8%), confirming that animal‐associated *C. albicans* populations represent a substantial and still largely unexplored reservoir of genetic diversity (Rhimi et al. 2025). Nevertheless, our study has some limitations that should be acknowledged. Although it represents one of the largest molecular investigations of wildlife‐derived *C. albicans* isolates to date, the sample size remains relatively limited and encompasses only a restricted range of host species. Moreover, the marked genetic heterogeneity observed, together with the limited number of isolates per host species, precluded robust species‐specific analyses. Therefore, future studies involving larger sample sizes and a broader taxonomic diversity of wildlife hosts will be essential to determine whether *C. albicans* population structure varies among host species.

Phylogenetic analyses of MLST data showed that animal isolates were distributed across multiple established *C. albicans* clades (1, 3, 7, 10, 11, 12, 14, 15 and 17) and often clustered with human clinical isolates (Figure 3). Nevertheless, although no definitive evidence for host‐specific lineages was found, a certain degree of genetic separation between human and animal isolates was detected by nearest‐neighbour analysis, consistent with previous observations reported in other studies (Edelmann et al. 2005; Jacobsen et al. 2008). However, despite the lack of absolute genetic separation, recent studies indicate that animal isolates often belong to different clades or display a different clade distribution compared to human isolates (Domán et al. 2021; Rhimi et al. 2025). For example, avian isolates often show a distinct subpopulation, frequently appearing within Clade 15 or other minor MLST clades, separated from the main human‐associated Clade 1 (Domán et al. 2021). Interestingly, Wrobel et al. (2008) demonstrated significant differences in clade distributions between human and wildlife isolates from the same geographic area (Illinois, USA), supporting the isolation of the fungal population between the two groups, despite evidence of limited strain transfer consistent with reverse zoonosis. Taken together, all these data show that human and animal *C. albicans* populations are not fully segregated by host, and although sharing common phylogenetic clades, they exhibit measurable host‐associated genetic differentiation, which should be further investigated using modern whole‐genome sequencing (Ropars et al. 2018) to better characterize patterns of population structure, and potential cross‐host transmission.

An interesting finding of this study is the isolation of numerous *C. albicans* isolates heterozygous at the *hwp1* locus, which clustered within clade 11 together with the rare homozygous 850 bp‐*hwp1* genotype identified here for the first time in animals (Morales‐López et al. 2025). *C. albicans* heterozygous *hwp1* isolates have been frequently reported in human clinical samples worldwide (Shan et al. 2014; Ngouana et al. 2015; Mucci et al. 2017; Fontecha et al. 2019; Pakshir et al. 2019; Nouraei et al. 2020; Salehipour et al. 2021; Anaele et al. 2025; Morales‐López et al. 2025), and their presence is now also confirmed in animal hosts. Nevertheless, their clinical relevance remains unclear, as no significant differences have been observed in biofilm formation (Pakshir et al. 2019) or in exoenzyme production, including phospholipase, hemolysin and proteinases (Nouraei et al. 2020). Interestingly, Orsi et al. (2014) showed that, although the *hwp1* genotype does not influence *C. albicans* biofilm production, it affects fungal morphology and susceptibility to microglial cells, highlighting the need for further studies to clarify the impact of the different polymorphisms currently identified at the *hwp1* locus (Ngouana et al. 2015; Morales‐López et al. 2025).

In conclusion, this study demonstrates that similar *C. albicans* genotypes are present in both animals and humans when analysed using different methods or genetic markers (ABC typing, MLST, *Hwp1*‐typing), suggesting potential cross‐transmission. However, the direction of transmission still remains unclear, with both animal‐to‐human and/or human‐to‐animal pathways being hypothesized (Edelmann et al. 2005; Wrobel et al. 2008). Therefore, further large‐scale genomic studies involving *C. albicans* isolates from diverse wild and domestic animal species and different geographic regions are needed to improve our understanding of the ecology, evolution and population biology of this important fungal pathogen within a One Health framework.

## Author Contributions

**Lucilla Lo Re:** investigation, methodology, formal analysis, visualization, writing – original draft. **Silvia Crotti:** methodology, resources, formal analysis, validation, writing – original draft. **Letterio Giuffrè:** formal analysis, software, validation, writing – original draft. **Andrea Bonomo:** data curation, software, validation. **Deborah Cruciani:** investigation, methodology, visualization. **Alice Ranucci:** investigation, data curation, validation. **Ida Pernice:** formal analysis, data curation, writing – original draft. **Carla Lo Passo:** formal analysis, visualization, writing – original draft. **Giuseppe Criseo:** supervision, writing – original draft, writing – review and editing. **Orazio Romeo:** conceptualization, project administration, supervision, funding acquisition, writing – original draft, writing – review and editing.

## Funding

This work was supported by the University of Messina, Italy (grant CT_GOM_2024_2025 to O.R.) and in part by the Italian Ministry of Health (grant RC002/2021 IZSUM‐‘WildSENTINEL’).

## Conflicts of Interest

The authors declare no conflicts of interest.

## Supporting information


**Table S1:** Detailed metadata for *C. albicans* isolates from Italian wildlife, including collection details, host information and phenotypic and genetic characteristics.

## Data Availability

All MLST data generated in this study are included in the article and are publicly available in the *C. albicans* PubMLST database (https://pubmlst.org/calbicans).
